# Confirmatory and validation studies on experimental self-efficacy scale with applications to multiple scientific disciplines

**DOI:** 10.3389/fpsyg.2023.1154310

**Published:** 2023-04-18

**Authors:** Vysakh Kani Kolil, S. U. Parvathy, Krishnashree Achuthan

**Affiliations:** Center for Cybersecurity Systems and Networks, Amrita Vishwa Vidyapeetham, Kollam, Kerala, India

**Keywords:** experimental self-efficacy, scale validation, confirmatory factor analysis, validity, laboratory, education

## Abstract

Laboratory education is essential for enhancing both the understanding of concepts and skills of students. A significant barrier to excelling in laboratory practices relates to a lack of self-efficacy. Being complementary to mainstream theoretical learning, the contribution of laboratory education to impart knowledge and hands-on proficiency is often under-represented. The aim of this research was to validate a novel experimental self-efficacy (ESE) scale and explore its relationship with laboratory outcomes, using gender and year of study as mediating variables. ESE refers to students' faith in their potential to carry out experiments and achieve desired outcomes in laboratory settings. When students possess strong ESE, they display more confidence in their abilities, accept tasks of greater difficulty levels, and have more tenacity to overcome obstacles. Data from 1,123 students were analyzed, focusing on the link between ESE constructs and laboratory experiments. Results indicated that ESE had a significant impact on laboratory performance in students of both genders and was related to factors such as laboratory hazards, conceptual understanding, the sufficiency of laboratory resources, and procedural complexities. The study affirms the validity and applicability of the ESE-scale to not only multiple disciplines such as chemistry, physics, and biology but also its relationship with students' academic outcomes in laboratories.

## 1. Introduction

In a technology and innovation-intensive society, laboratory work is expected to help students build their analytical and problem-solving aptitudes. In science and engineering courses, gaining hands-on experience through laboratories is an inevitable and critical aspect of student learning (Achuthan et al., 2021; Srinivasa et al., 2021; Diwakar et al., 2023). Especially in STEM (science, technology, engineering, and mathematics), there is an emphasis on using laboratory experimentation as gateways to visualize concepts and applications. The concept of “learning by doing” (Ilhan et al., 2015) is well-established. It is well-documented that students conduct experiments effectively if they have pre-existing knowledge on experimental procedure and set-up (Brüggemann and Bizer, 2016). Further, they require a well-maintained experimental set-up, seasoned laboratory instructors, and qualified faculty (Ernst, 1983). Through hands-on practice in laboratory work, students understand how to handle storage and hazardous materials (Artdej, 2012). They also learn, by doing, the importance of safety guidelines and labels. However, the outcomes of learning by doing in laboratory learning contexts are less explored.

In physical laboratories, the drawback is that many pre-designed laboratory experiments fundamentally give a reductive learning experience confined to observation and learning from plotted results. Oversimplified experiments could look like merely taking readings after placing a few dials. For example, in a regular materials testing laboratory, students learn about the characteristics of the material from the output graph after analysis is performed. They do not learn while the analysis is taking place. Therefore, we can expect students to know little about material retaliation or the material's stress state for the duration of the test. Their knowledge will be limited to safety procedures and analysis (Vergara et al., 2017). Critical thinking and deep learning are disadvantaged as a result (Kapilan et al., 2021).

When the number of enrolled students increases and cost of procuring equipment also increases, chances to perform experiments become constrained (Srinivasa et al., 2021) due to resource limitations. Some studies concur that the status quo in India is ever-growing student numbers in classrooms of government schools and colleges that are poorly funded and ill-equipped to handle student needs (Kulshreshtha et al., 2022). This affects student learning greatly. Resource insufficiency has a real impact on students. For example, if the average classroom capacity of Bachelor of Science program undergraduate classes is approximately thirty students per class, it is difficult to provide equipment such as one compound microscope per student. The norm at government colleges is sharing of laboratory equipment or taking turns to use it. The literature puts forth that the result is degradation in learning quality as these situations culminate in student dissatisfaction, loss of interest in learning material, and copying of other students' laboratory observations (Kulshreshtha et al., 2022).

In the last decade, there has been growing evidence that laboratory work has the capability to boost student interest and improves their abilities in science subjects (Hofstein and Mamlok-Naaman, 2007; Petritis et al., 2022). However, it is impossible to demonstrate most theoretical concepts because of the above mentioned scarcity of laboratory equipment, trained professionals, constricted time allotted for practical laboratory experimentation, and so on.

Researchers have been exploring ways to boost the quality of learning and laboratory outcomes but laboratory intensive courses, assessment of students' learning poses more of a challenge (Qu and Lu, 2012). The difficulty in assessing laboratory work also stems from the fact that although the challenges students face in laboratories is discussed in the literature, methods to overcome them are not as established. Some methods listed in the literature include developing alternate mediums for students to learn content and laboratory skills (Raman et al., 2015; Chowdhury et al., 2019; Raman et al., 2022). Aside from hard skills in the subject, student work in laboratories is improved only when their self-efficacy or self-confidence in their abilities improves. However, there is a lack of holistic instruments that assess student self-efficacy in laboratory contexts. Collecting data on student self-efficacy is a crucial and significant step toward improving the quality of laboratory learning in STEM education. Developing countries risk the declining availability of STEM practitioners if the situation persists due to the exacerbated constraints they face.

There are several difficulties in science laboratory education that can affect students' self-efficacy, including lack of access to equipment and resources, inadequate training and support for teachers, and lack of opportunities for hands-on learning. According to the literature (Bowen, 1999; Kamaruddin et al., 2019), the major difficulties in science laboratory education are: (1) safety: laboratories can be dangerous places if proper safety protocols are not followed. This can lead to students feeling anxious or uncomfortable in the lab, which can negatively impact their learning and self-efficacy, (2) procedural complexity: experiments can be complex and require a high level of attention to detail. This can be challenging for students who are new to the subject or who have difficulty with hands-on activities, (3) limited resources: many schools and universities have limited resources, which can make it difficult to provide students with the necessary equipment and materials for experiments. This limits students' ability to engage in hands-on and inquiry-based learning. This can negatively impact their self-efficacy by making them feel less capable of understanding and applying scientific concepts, (4) difficulty in conceptual understanding: students might find it difficult to understand the experiment and its objectives, which can lead to confusion, frustration, and low self-efficacy, (5) limited time: laboratory classes usually have limited time allotted in their curriculum, which can add pressure on their completion times and limit reflective thinking in the laboratory to understand the results fully, (6) inadequate training: another difficulty is that technicians or supervisors may not have adequate training to effectively communicate laboratory instructions. This can lead to a lack of confidence amongst the students resulting in their low self-efficacy, and (7) lack of opportunities: Additionally, students may not have enough opportunities to engage in hands-on and inquiry-based learning in the laboratory setting, which can negatively impact their self-efficacy by making them feel less competent in applying scientific concepts. All of these difficulties can negatively impact students' self-efficacy. Students feeling confident in their abilities are more likely to achieve success by fully participating in the learning process.

A key predictor of facets of academic success i.e., academic motivation (Kausik and Hussain, 2021; Ariff et al., 2022), self-regulation (Arcoverde et al., 2022; Kryshko et al., 2022), and academic achievement (Affuso et al., 2022; Jindal and Sharma, 2022) constitutes self-efficacy. Although the term refers to students' cognitive competence to do a particular activity (Bandura et al., 1999), it is the yardstick to analyze students' performance and diligence (Heslin and Klehe, 2006).

In the last two decades, facets of self-efficacy have been studied extensively across disciplines, both academic and non-academic (Flores, 2015; Kola and Sunday, 2015; Mieder, 2016). While educators are primarily concerned with imparting content and skills, self-efficacy research posits that mere possession of knowledge and skills does not necessarily ensure that students will apply them (Artino, 2012). Therefore, students' self-efficacy related to experiments has come under scrutiny in recent times (Shaari et al., 2014).

Unlike other quantifiable variables, self-efficacy is not calculated in an easy way. Self-reported answers to survey questions may generally assess the self-efficacy of a person (Fakhrou and Habib, 2022; Huang, 2022; Mubarrak et al., 2022). In the past, researchers have relied on general, multiple-scale self-efficacy determinants available around the world to gauge academic performances (Multon et al., 1991; Tsai et al., 2021; Dixon and Ward, 2022). Determinants of self-efficacy are most telling and useful for predicting areas with an absence of self-efficacy. Alternatively, they are useful when aimed at analyzing particular student behaviors, work, or goals (Heslin and Klehe, 2006). There have also been other instruments designed for evaluating students' self-efficacy regarding hypothetical academic behaviors (Wood and Locke, 1987; Pintrich and De Groot, 1990; Bong, 2004; Sander and Sanders, 2009; Boulden et al., 2021; Pumptow and Brahm, 2021; Karaoglan Yilmaz, 2022; Nasaif et al., 2022; Sánchez-Escobedo, 2022; van Zyl et al., 2022). Even though some studies have conducted research in self-efficacy in higher education, there is insufficient focus on students' self-efficacy in laboratory work. There is a need for instruments specifically to measure students' belief in themselves to perform a variety of essential laboratory tasks (Gore, 2006).

Some studies explore students' self-efficacy in chemistry, for example (Dalgety et al., 2003; Aydın and Uzuntiryaki, 2009; Uzuntiryaki and Aydın, 2009). However, these instruments are limited due to the lack of scalability or use in laboratory contexts (Alkan et al., 2016). To our knowledge, one prior instrument exists on experimental self-efficacy (Alkan et al., 2016), which took into account the psychomotor and self-efficacy beliefs of students in chemistry laboratories. However, that instrument is missing other predictors of laboratory performance, such as measuring student anxiety in performing tasks due to laboratory hazards or the absence of sufficient resources. The lack of a comprehensive experimental self-efficacy instrument has contributed to the lack of improvement in higher education quality in STEM courses. These gaps are addressed by our study, which has designed and validated a comprehensive experimental self-efficacy instrument.

Up till the pandemic, very few higher education institutes were designing and implementing platforms to establish educational e-strategies (Subashini et al., 2022). However, since the pandemic, developing countries have made progress to overcome their logistical, economical, and infrastructural barriers to conducting laboratory education. After the provision of laboratory education, the quality of laboratory education made available to students is, thus, coming under scrutiny post-pandemic. Thus, increasing and assessing students experimental self-efficacy will be the natural next step.

This article is organized as follows: we conducted the exploratory factor analysis and item analysis of the ESE-scale using an undergraduate students sample. Then the four-factor model of the ESE-scale was confirmed in the multi-discipline area. Thereafter, we analyzed the experimental self-efficacy in the laboratory outcome using gender and the year of study as mediating variables. We have summarized the evidence on the reliability and validity of the ESE-scale in laboratory education literature in this publication.

### 1.1. Self-efficacy instruments

The literature presents some instruments that have been designed to capture self-efficacy. The most commonly used scale is the General Self-Efficacy Scale (GSE) (Schwarzer and Jerusalem, 1995), which comprises 10 items that evaluate students' general sense of self-efficacy across various contexts. This measure is commonly used across different cultures and fields, including health (Hu et al., 2020), work (Xanthopoulou et al., 2009), and education (Schwarzer and Hallum, 2008). Another widely used measure is the Specific Self-Efficacy Scale (SSE) (Sherer et al., 1982) which calculates self-efficacy in particular domains or tasks, and it is applicable in various fields such as health, education, and sports. Other self-efficacy measures are applicable in particular populations only. For example, the Self-Efficacy for Managing Chronic Disease Scale (SEMCD) assesses self-efficacy in managing lifelong diseases, such as heart disease or diabetes (Hudon, 2014) only. Another context-specific scale is The Multidimensional Scale of Perceived Social Support (MSPSS) which gauges perceived social support (Zimet et al., 1988). Depending on the field or population of interest, it is important to select an appropriate measure of self-efficacy and be aware of its limitations.

### 1.2. Experimental self-efficacy scale

**Figure 1:**
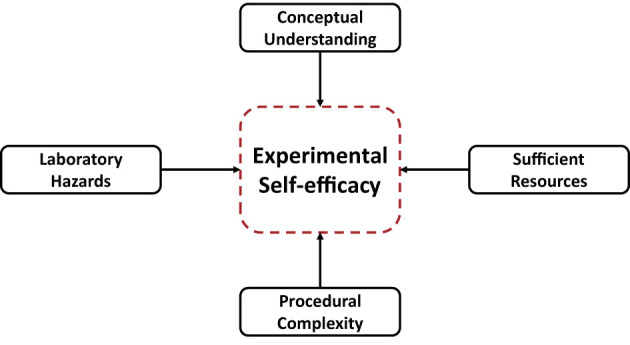
Experimental self-efficacy and its factors. Adapted from Kolil et al. (2020).

Experimental self-efficacy (ESE) is an essential factor in students handling curriculum with experiments (Kolil et al., 2020). According to Kolil et al. (2020), experimental self-efficacy encapsulates students' belief in their abilities to perform laboratory activities. Experimental self-efficacy is often studied through the lens of social cognitive theory, which suggests that individuals' beliefs in their abilities can impact their motivation, goal-setting, and task performance. Four primary factors impacting experimental self-efficacy were determined and categorized into two theoretical aspects: cognitive activity and physical activity. Namely, laboratory hazards (LH), conceptual understanding (CU), the sufficiency of resources (SR), and procedural complexity (PC) (Figure 1). The four factors were identified based on literature, feedback from instructors, and through online surveys and in-person interviews with students.

Cognitive activity includes CU and PC, and physical activity involves anxiety caused by LH or SR. Laboratory hazards refer to any potential accidents that could occur in the laboratory, such as accidentally breaking apparatus or spilling chemicals. Conceptual Understanding refers to the student's knowledge of the experiment's theoretical foundation. This includes understanding the concepts and principles that are being tested in the experiment. Sufficiency of resources, on the other hand, refers to the adequacy of laboratory apparatus (such as beakers, burettes, pipettes), instruments (such as flamephotometer, calorimeter), and instructors to perform the experiments. Procedural complexity refers to the difficulties faced by students in calculating arithmetically and executing the experiment steps. This includes understanding the mathematical equations and calculations that are required to be done in order to complete the experiment, as well as understanding the steps that need to be taken in order to complete the experiment. After the development of a reliable instrument for measuring ESE, the study demonstrated the positive effects of virtual laboratories (VL) on students with low ESE scores. Statistical analysis showed a significant increase in conceptual understanding in students. Further, procedural complexity reduced anxiety caused by laboratory hazards, or lack of resources was alleviated.

### 1.3. Effect of gender on self-efficacy

Research has shown that there are some gender differences in self-efficacy in the academic field. Studies have found that, on average, females tend to have lower self-efficacy than males in certain subject areas, such as mathematics and science. However, other studies found no gender differences or even females having higher self-efficacy than males in certain fields. The studies which characterize the link between gender and science self-efficacy (Li and Singh, 2021; Bhati et al., 2022; Smith et al., 2022), have found that males reported possessing greater science self-efficacy than females (Anderman and Young, 1994; Lee et al., 2022). However, it seems to be discipline specific. For example, females are more confident in performing tasks in the field of life sciences while males seem to fare better in the physical sciences(Wang et al., 2016). To embellish science, self-efficacy is related to students' areas of scientific expertise too. Physics and chemistry majors have higher self-efficacy in the physical sciences compared to students who majored in engineering and biology (Bodner et al., 2015). Multiple factors contribute to these gender differences in self-efficacy, including socialization, stereotypes, discrimination, and access to role models. For example, girls and women may receive less encouragement and support than boys and men in certain subject areas, and they may be more likely to encounter stereotypes and biases that suggest they are not as capable as boys and men in those areas. This can lead to girls and women having lower self-efficacy in those areas (Wigfield and Eccles, 2002; Huang et al., 2019). On the other hand, studies have also suggested that interventions aimed at reducing gender gaps in self-efficacy and performance in STEM fields have had some success. These interventions have included providing opportunities for girls and women to engage in hands-on activities, providing role models, and addressing stereotypes and biases.

### 1.4. Research objectives

The following research questions were examined in this paper:

- Validate the four-factor experimental self-efficacy scale using chemistry students sample from higher education institutes.
- Test the reproducibility of the four-factor ESE scale on the students enrolled in physics and biology subjects.
- Test the relationship between ESE and Laboratory outcome of students.
- Test the mediation effect of year of study and gender on the ESE-Laboratory outcome relationship.

## 2. Materials and methods

### 2.1. Participants

The undergraduates (*N* = 1,123) participating in this study were from 26 tertiary education colleges in 11 states (Kerala, Tamil Nadu, Karnataka, Maharashtra, Andhra Pradesh, Gujarat, Rajasthan, Telangana, Pondicherry, Punjab, and West Bengal) in India. The participants were then categorized into two groups (Group 1 and Group 2). Group 1 had all chemistry students (*N* = 684), whereas Group 2 (*N* = 439) included students from the physics and biology disciplines (Table 1). Out of 684 participants, 49.1% were male students, and 50.8% were female students in Group 1. In Group 2, 37.4% were male students, and 62.6% were female students. All the participants were in the age brackets of 21–24 years (SD = 0.958). Out of 26 institutes, 11 were engineering colleges, and 15 were arts & sciences colleges. The survey was conducted during the academic year 2021–22.

**Table 1:** Demography of the participants.

**Category**		**Group 1**	**%**	**Group 2**	**%**
*N*		684		439
Gender	Male	336	49.1%	164	37.4%
	Female	348	50.8%	275	62.6%
State	Kerala	148	21.6%	220	50.1%
	Tamil Nadu	394	57.6%	139	32.0%
	Karnataka	9	1.3%	–	–
	Maharashtra	19	2.8%	–	–
	Andhra Pradesh	12	1.8%	–	–
	Gujarat	5	0.7%	–	–
	Rajasthan	1	0.1%	1	0.2%
	Telangana	57	8.4%	1	0.2%
	Pondicherry	1	0.1%	–	–
	Punjab	37	5.5%	77	17.5%
	West Bengal	–	–	1	0.2%
Year of study	First year	459	67.1%	294	66.8%
	Second year	106	15.5%	80	18.2%
	Third year	119	17.3%	65	15.0%
# Institute		23		26
	Engineering	9	39.1%	11	42.3%
	Arts and sciences	14	60.9%	15	57.7%
	Other	6	26.1%	6	23.1 %

### 2.2. Measures

Students' experimental self-efficacy was measured using the “Experimental self-efficacy (ESE)” questionnaire developed by Kolil et al. (2020). This scale consists of 12 items that are organized into four different factors: (1) conceptual understanding (CU), (2) laboratory hazards (LH), (3) procedural complexity (PC), and (4) sufficiency of resources (SR). Items 1–3 belong to CU, 4–6 to LH, 7–9 to PC, and 10–12 to SR. The response format for the questionnaire uses a Likert scale with options ranging from 1 to 5, with 1 indicating “strongly disagree,” and 5 indicating “strongly agree.” The complete questionnaire is included in Appendix A. The Likert scale format allows for a more nuanced understanding of a person's opinion than a simple yes or no answer.

The measures of students' laboratory performance (Lab score) were obtained from the laboratory instructors of 26 participating institutes. Performance in a laboratory was determined by the grades given to students at the end of the semester by the instructor. These grades were based on a final laboratory examination consisting of physical laboratory experimentation. These final examinations were conducted on different dates based on the availability of instructors. All the participating students from the same institutes attended the examination on the same day. A common rule was followed for the examination (four experiments) by all the institutes, and the scores of final grades ranged from 10 to 20 (maximum five grades of one experiment). The selection of four experiments is based on the curriculum that the institute follows.

During the online survey, information about the students' demographic characteristics, including their age, gender, year of study, type of institute they attend (e.g., engineering, arts, and sciences), and location (state), was gathered.

### 2.3. Procedure

**Figure 2:**
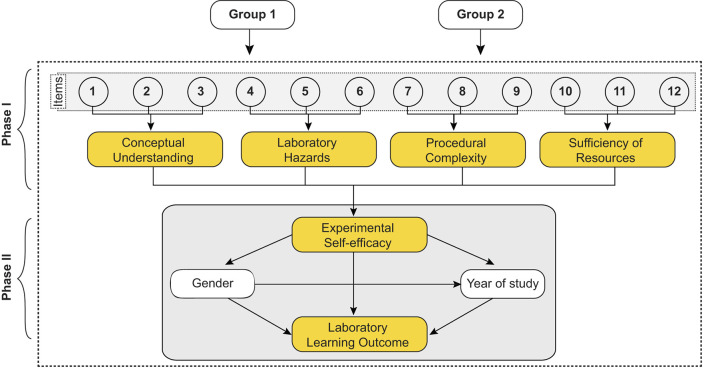
Study design.

The online survey was conducted using Google Forms platforms. The survey form was sent to 26 randomly selected institutions, and responses were collected from 1,359 students from the chemistry, physics, and biology disciplines. The responses were then compiled and analyzed using IBM SPSS^©^ statistics 20 software. During the analysis, 234 duplicate responses were identified and removed. Each student was given a unique identifying code for the purpose of tracking their progress and performance. The information provided by the institutions or organizations was kept anonymous to protect the privacy of the participants. The study design of the study was provided in Figure 2.

In the first phase of the study (phase I), the validity of the ESE questionnaire was checked using confirmatory factor analysis (CFA) and structural equation modeling (SEM). The model includes all the participants.

In the second phase of the study (phase II), the effect of ESE on students' laboratory learning outcomes was analyzed using linear regression analysis. The student's gender and year of study were used as the mediating factors for the analysis. The effect was also analyzed separately with Group 1 and Group 2 participants.

### 2.4. Statistical methods

Scale validation involved performing several analyses. Exploratory Factor Analysis (EFA) was carried out by the Principal component method with Varimax rotation. Kaiser normalization was used to determine the number of factors. Participants' responses were divided into three groups by year of study (first year, second year, and third year), two groups by disciples (Group 1: chemistry and Group 2: physics and biology), and two groups by gender (Male and Female).

### 2.5. Data analysis

#### 2.5.1. Preliminary analysis of the data

We conducted an examination of different statistical measures for all items that were included in the survey. These measures include the mean, standard deviation (SD), skewness and kurtosis. The measure skewness shows the lack of symmetry in the data distribution while kurtosis shows the degree of peakedness or flatness of the distribution.

#### 2.5.2. Construct validity

Based on 1,123 responses gathered through the experimental self-efficacy scale, validity and reliability analysis was conducted. Using Cronbach's alpha (α) internal consistency coefficient, the reliability of the whole scale and factors was calculated. The four factors of the ESE-scale were supposed to assess the same feature. Thus, Pearson correlation coefficients (*r*) were calculated. We adhered to the recommendations of Schmitt et al. (2018) to gather evidence of the construct validity of the scale. The “best” factor structure can be retained using methods such as exploratory factor analysis (EFA) or confirmatory factor analysis (CFA). The main drawback of EFA is that results are difficult to reproduce with various samples, whereas CFA produces biased factor loadings and correlations since it demands that cross-loadings in the non-target factors be zero (Garn et al., 2019). Therefore, in view of the above information and our data, we test the four-factor model *via* CFA in the present study.

The descriptive data analysis, EFA, reliability, and validity analysis were performed using IBM^©^ SPSS^©^ Statistics 20 and CFA was performed using IBM^©^ SPSS^©^ AMOS 26 Graphics software.

## 3. Results

### 3.1. Preliminary analysis of the data

The scores on the ESE scale for different items show varying means, with item Q9 having the lowest mean at 3.71 and item Q10 having the highest mean at 4.05. The standard deviations (SD) of these scores also vary greatly, starting from a low of 1.017 for item Q7 to a high of 1.253 for item Q1. The skewness, which indicates the distribution's asymmetry, also differs among the scores, with item Q10 having the most negative skewness at –1.215 and item Q9 having the least negative skewness at –0.769. The kurtosis values range from –0.175 (Q1) to 1.105 (Q6) which provides information about the distribution's shape. The descriptive statistics results are tabulated in Table 2.

**Table 2:** Mean, standard deviation, Pearson correlation with the total ESE score and critical ratio of the ESE-scale items (*N* = 1,123).

**Item**	**Mean**	**SD**	**Skewness**	**Kurtosis**	**Correlation with the**		**Critical ratio**
					**total ESE score**	**subscale total score**
Q1	3.84	1.253	–0.919	–0.175	0.468^**^	0.886^**^	14.548^**^
Q2	3.99	1.250	–1.160	0.279	0.441^**^	0.881^**^	12.650^**^
Q3	3.90	1.218	–1.059	0.199	0.467^**^	0.888^**^	14.262^**^
Q4	3.99	1.075	–1.119	0.814	0.667^**^	0.882^**^	23.932^**^
Q5	3.96	1.056	–1.085	0.857	0.634^**^	0.880^**^	22.458^**^
Q6	4.02	1.031	–1.153	1.105	0.605^**^	0.877^**^	19.191^**^
Q7	3.90	1.017	–1.003	0.879	0.628^**^	0.872^**^	22.502^**^
Q8	3.82	1.057	–0.831	0.285	0.616^**^	0.880^**^	23.480^**^
Q9	3.71	1.125	–0.769	0.026	0.592^**^	0.890^**^	22.092^**^
Q10	4.05	1.086	–1.215	0.973	0.617^**^	0.882^**^	22.004^**^
Q11	3.99	1.073	–1.142	0.925	0.576^**^	0.863^**^	19.849^**^
Q12	3.90	1.236	–1.046	0.138	0.591^**^	0.891^**^	20.360^**^

^**^ *p* < 0.01.

### 3.2. Classical test theory item analysis

A Pearson correlation (*r*) was conducted to examine the relationship between each item on the ESE questionnaire with both the total scores of the ESE-scale and its subscales. The results of the correlation analysis revealed that the *r* between each item in the ESE-scale and the overall ESE-scale score was between 0.441 and 0.667. Moreover, the correlation between each item in the ESE-scale and the subscale scores ranged from 0.863 to 0.891. These correlation coefficients were used as the basis for the item analysis, along with the critical ratio (CR) values (Table 2). To further examine the discriminability of each item, the participants were divided into two groups based on the scores, i.e., the high-score group and the low-score group (Peng et al., 2021). Each participant was ranked based on their total score by arranging them in descending order of total score. The participants who belonged to the top 27% (*N*_*high*_ = 1123*(27/100) = 303) ranked position were considered into the high-score group, and those in the bottom 27% (*N*_*low*_ = 1123*(27/100) = 303) ranked position were considered into the low-score group. That is, if *Rank* < = 0.27*N, the participant belongs to the high-score group, and if *Rank*> = (1 − 0.27)*N+1, the participant belongs to the low-score group. Each item between high-score and low-score groups was compared using an independent *t*-test. The results of the CR-test revealed that there was a statistically significant difference in each item's score in the ESE-scale between the two groups (high and low-score groups) (*p* < 0.01), demonstrating the great discriminability of all items.

### 3.3. Internal consistency of the ESE scale

The Cronbach's α value was 0.810 for the whole sample (*N* = 1,123) and ranges from 0.851–0.861 for the subscales (factors) measuring conceptual understanding (α = 0.861), procedural complexity (α = 0.854), laboratory hazards (α = 0.855) and sufficiency of resources (α = 0.851) (Table 3). The Spearman correlation (ρ) between individual items and the total score of the ESE instrument were significant and positive in the whole sample. The correlations of individual items with the total score range from 0.380–0.650. These results indicated an acceptable level of internal consistency (Table 3). The split-half method was also used to assess the internal consistency of the ESE-scale by dividing the items of each factor into two halves and comparing the scores on each half. The first half of the factor consists of the first two items, and the second consists of the remaining one items (Table 3). The results confirm that the split-half coefficient (Spearman-Brown Coefficient) values are above the critical value of 0.70, suggesting the high reliability of the scale.

**Table 3:** Results of the exploratory factor analysis (EFA) of group 1.

**Item**	**F 1 CU**	**F 2 LH**	**F 3 PC**	**F 4 SR**	**Cronbach's Alpha (α)**	**AVE^b^**	**Composite Reliability**	**Item-score correlation (ρ)**	**Alfa Cronbach global split-half coefficients**
Q1	0.884							0.426^*^	
Q2	0.870				0.861	0.775	0.912	0.380^*^	0.868
Q3	0.887							0.424^*^
Q4		0.812						0.650^*^	
Q5		0.843			0.854	0.705	0.878	0.602^*^	0.854
Q6		0.864						0.564^*^
Q7			0.845					0.613^*^	
Q8			0.852		0.855	0.734	0.892	0.618^*^	0.859
Q9			0.873					0.599^*^
Q10				0.810				0.580*	
Q11				0.851	0.851	0.702	0.876	0.531^*^	0.853
Q12				0.851				0.527^*^
Variance	1.541	1.111	1.144	1.289.
Cronbach's α of ESE = 0.810.									
KMO^a^ measure of sample adequacy = 0.819.									
% of cumulative variance explained = 77.944.									
Spearman-Brown coefficient = 0.844.									

^*^significant at *p* < 0.01.

^a^Kaiser-Meyer-Olkin.

^b^Average variance extracted.

To examine the underlying structure of the data and confirm the underlying patterns or relationships that exist within the variables of the data, an exploratory factor analysis was conducted on the data of complete participants (*N* = 1,123). The Kaiser-Meyer-Olkin (KMO) sample adequacy test was used to evaluate the suitability of the scale for factor analysis, and the obtained KMO value of 0.819 indicates the scale is appropriate for factor analysis (Tabachnick and Fidell, 2001). The scree plot, which is a graphical representation of the eigenvalues of each factor, confirmed that four factors had an eigenvalue >1, indicating that these four factors were the most important. The factor loadings, which indicate the degree to which each item is associated with each factor, exceeded 0.80 (*p* < 0.01) for all items under each of the four factors, and these factors could explain 77.944% of the variance. These results are tabulated in Table 3. Factor loading values were used to calculate the average variance extracted (AVE) and composite reliability of the factors. The results showed that the AVE values of all the factors (CU = 0.775, LH = 0.705, PC = 0.734, and SR = 0.702) were above the critical value of 0.50, indicating good convergent validity. Similarly, the composite reliability (CU = 0.912, LH = 0.878, PC = 0.892, and SR = 0.876) were above the critical value of 0.70, providing further evidence of the scale's validity (Hair et al., 2006; Izogo, 2016).

### 3.4. Construct validity

**Figure 3:**
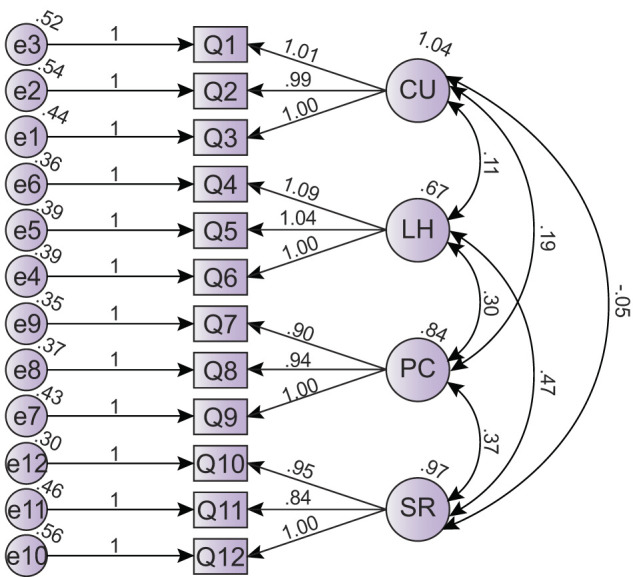
Confirmatory factor analysis (*N* = 1,123).

The proposed model was analyzed using confirmatory factor analysis (Figure 3) and observed that the probability that a four-factor CFA of the model was *p* < 0.001 (χ^2^ = 124.018, df = 48). The list of model fit indices and their corresponding threshold values were tabulated in Table 4. The results indicate the model fit indices of the model fall within acceptable ranges. The fit indices values of the four-factor model, AGFI (adjusted goodness-of-fit index) = 0.970 [threshold value = >0.8 (Bagozzi and Yi, 1988)], RMSEA (root mean square error of approximation) = 0.038 [threshold value = <0.08 (Chau and Hu, 2001)], NFI (normed fit index) = 0.982 [threshold value = >0.9 (Browne et al., 1993)], CFI (comparative fit index) = 0.989 [threshold value = >0.9 (Chin and Todd, 1995; Hair, 1998)] and the ratio of χ^2^(113.666) to degrees of freedom (df= 48) = 2.584 [threshold value = < 3 (Bagozzi and Yi, 1988)], had better values than the thresholds. Thus, the CFA results revealed that the measurement model fit with the data collected for the study. The outcome of the confirmatory factor analysis of the ESE-scale confirms its four-factor structure and provides evidence for the scale's construct validity, which refers to the extent to which the scale measures what it is intended to measure.

**Table 4:** Fit indices of four-factor model (*N* = 1,123).

**Model**	χ^**2**^		**χ^2^/df**	**AGFI^a^**	**RMSEA^b^**	**CFI^c^**	**NFI^d^**
	**Value**	**df**					
Four-factor model	124.018	48	2.584	0.970	0.038	0.989	0.982

^a^Adjusted goodness-of-fit index.

^b^Root mean square error of approximation.

^c^Comparative fit index.

^d^Normed fit index.

### 3.5. Correlation of ESE factors with laboratory score

The correlation between ESE factors (CU, LH, PC, and SR) with laboratory scores was analyzed using Pearson correlation (*r*) analysis. The analysis suggests that all the factors in both Group 1 and Group 2 are highly correlated (*p* < 0.01) with the laboratory scores (Table 5). The high correlation between ESE factors and laboratory scores suggests the importance of analyzing ESE in laboratory classrooms.

**Table 5:** Pearson correlation (*r*) of ESE factors with laboratory outcome score.

**Group**		**CU**	**LH**	**PC**	**SR**
Group 1	Lab score	0.351^**^	0.445^**^	0.0388^**^	0.312^**^
Group 2	Lab score	0.802^**^	0.788^**^	0.850^**^	0.838^**^

^**^p < 0.01.

### 3.6. Relationship between ESE and laboratory scores

The regression model examines the relationship between the dependent variable (laboratory score) and several independent variables (gender, year of study, experimental self-efficacy, and their interactions) (Table 6). The analysis was conducted separately for Group 1 and Group 2. The analysis revealed that the models were significant (Group 1: *F*_6,677_ = 95.963, *p* < 0.001; Group 2: *F*_6,432_ = 565.427, *p* < 0.001), and the independent variables explained a significant amount of variance in the laboratory score (Group 1: *R*^2^ = 0.460; Group 2: *R*^2^ = 0.887).

**Table 6:** Linear regression model of Group 1 and Group 2.

**Variable**	***B***	**Std. error**	**β**	***t***	***p***
**Group 1**					
Intercept	14.686	0.091		161.233	0.000^*^
ESE	2.172	0.095	0.665	22.742	0.000^*^
Gender	–0.024	0.089	–0.008	–0.269	0.788
Year of study	–0.210	0.092	–0.070	–2.278	0.023^*^
ESE × Gender	0.213	0.096	0.066	2.223	0.027^*^
ESE × Year of study	–0.145	0.102	–0.043	–1.420	0.156
Gender × Year of study	0.132	0.093	0.042	1.409	0.159
**Group 2**					
Intercept	14.807	0.051		290.536	0.000^*^
ESE	2.748	0.050	0.975	54.425	0.000^*^
Gender	0.022	0.052	0.007	0.416	0.678
Year of study	–0.087	0.052	–0.028	–1.686	0.092
ESE × Gender	–0.266	0.052	–0.090	–5.091	0.000^*^
ESE × Year of study	0.130	0.054	0.040	2.425	0.016^*^
Gender × Year of study	0.074	0.052	0.023	1.409	0.159

^*^p < 0.05.

In Group 1, the standardized coefficient (β) for ESE is 0.665, indicating a moderate positive relationship between ESE and laboratory score. The coefficient (*B*) for gender is negative (–0.024), but it is not statistically significant (*p*> 0.05), indicating that gender does not have a significant relationship with laboratory scores in this group. The β for the year of study is –0.070, indicating a weak negative relationship between the year of study and laboratory scores (*p* = 0.023). The remaining coefficients involve interactions between the independent variables, such as ESE between Gender, ESE between the year of study, and Gender between the year of study. These coefficients reflect the effects of the combined influence of two independent variables on laboratory scores while controlling for the other independent variable. In the interaction variables, only gender shows a significant positive effect on the ESE of students (β = 0.066, *p* = 0.027) and the result suggests that gender indirectly affects the relationship between ESE and laboratory scores.

In Group 2, the standardized coefficient (β) for ESE is 0.975, indicating a strong positive relationship between ESE and laboratory scores (*p* < 0.001). The analysis suggests that the gender (*p* = 0.678) and the year of study (*p* = 0. 092) is not a valid predictor of laboratory scores in Group 2. While considering the interaction effects, gender (*p* ≤ 0.001) and the year of study (*p* = 0.016) show a significant effect on the ESE of students and which suggests that gender and year of study indirectly affect the relationship between ESE and laboratory scores. The collinearity statistics (tolerance (0.680–0.845) and VIF (1.183–1.470) suggest that there were no significant issues with multicollinearity in models 1 and 2.

## 4. Discussion

One's view of his or her own ability to carry out a specific task with a specific level of skill is known as self-efficacy. This parameter is important in that it is indicative of the student's preferences for learning. Theoretically, students would attempt to avoid tasks that are required for learning a subject if they do not feel capable of performing them (Bandura, 1986). According to Kiremit (2006), people who have high self-efficacy beliefs perform better and are in general happier as a result. Self-efficacy affects the mental processes and feelings that underpin classroom behavior (Pendergast et al., 2011). Self-efficacy is one of the key motivational factors that are essential for invoking student commitment and learning. Self-efficacy is examined in relation to how it could enhance behavioral change and cognitive immersion in the classroom (Linnenbrink and Pintrich, 2003). Previous research has shown that self-perception of efficacy influences the choice of activities, environmental settings, effort expenditure and persistence regardless of whether their judgments are faulty or accurate. Self-efficacy plays an important role in laboratory education as it can influence students' motivation, engagement, and performance in laboratory tasks. Students with high self-efficacy tend to approach laboratory tasks with greater interest, confidence, and persistence, which can lead to better performance and deeper learning. They also tend to set higher goals for themselves, choose more challenging tasks, and recover more quickly from setbacks or failures. Therefore, it's important to consider students' self-efficacy when designing laboratory activities, as well as when providing feedback, encouragement, and support to students. A number of strategies can be used to enhance students' self-efficacy in laboratory education, such as providing clear instructions, structuring tasks to match students' abilities, providing opportunities for success, modeling self-regulation, providing appropriate feedback, and training students to use self-regulatory strategies. A teacher or instructor also should also try to create a positive and supportive learning environment where students feel comfortable taking risks and learning from their mistakes, which can increase self-efficacy.

Data analysis from this work distinctly indicates the structural validity of the student's experimental self-efficacy scale. Given that the main objective of this study is the confirmatory analysis of the four-factor ESE scale in the chemistry laboratory classrooms and to check the applicability in other science disciplines like physics and biology, exploratory and structural equation modeling were used. A comprehensive set of analyses of items, reliability, and validity tests were performed. According to Paunonen and Hong (2010), there is a tendency for self-efficacy to be correlated with certain disciplines or fields of study. In other words, an individual's level of self-efficacy is often related to their chosen area of expertise or specialization. ESE relates to the laboratory experimentation sphere and can successfully predict laboratory performance and academic satisfaction well (Kolil et al., 2020). The ESE-scale is, therefore, an exceptional instrument for assessing ESE and conceptual understanding.

First, the item analysis results showed a significant, positive association between each item score and the final score. There were significantly distinguishable differences between the high-score and the low-score groups for each item. These results verified the high item quality of the ESE-scale. A four-dimensional model resulted from the exploratory factor analysis, and the loads on all item factors were above 0.80. The clarity in the item content and its high decipherability could be ascertained from the fact that the principal component analysis explained 77.944% of the variance (Peng et al., 2019). With regard to reliability, the original ESE-scale had a reliability coefficient (α) of 0.86, and each factor had a reliability value above 0.7 (CU = 0.76, LH = 0.82, PC = 0.89, and SR = 0.72) (Kolil et al., 2020). In the current analysis, the ESE-scale had an Alfa Cronbach global split-half coefficient of 0.844 (Spearman-Brown Coefficient), which indicates that the scale is reliable. The internal consistency coefficient was found to be 0.810, and this suggested that the ESE scale had reasonably good stability and consistency (Rigotti et al., 2008). The high reliability of each of the factors (CU = 0.861, LH = 0.854, PC = 0.855, and SR = 0.851) was also verified by analyzing a different set of samples. Confirmatory factor analysis was done to complement the results from the exploratory factor analysis and verify the expected theoretical structure. The confirmatory factor analysis results showed that the four-factor model of the ESE scale fit the data well, suggesting that the ESE scale had high construct validity.

The four-factor ESE model showed the best fit; thus, the four-factor ESE structure was retained. The AVE (average variance extracted) was CU = 0.775, LH = 0.705, PC = 0.734, and SR = 0.702 (that are comfortably higher than the critical value of 0.50), suggesting good convergence of the scale (Izogo, 2016). With the extensive analysis done here and additional comparisons made to the literature, the four-factor model for ESE scale developed is established as a reliable and valid measurement tool. When the findings of the research and those in the literature are compared, the four-factor structure of the experimental self-efficacy scale is verified, and a reliable and valid measurement tool is developed. Similar to Kolil et al. (2020), where the authors reported the Kaiser-Mayer-Olkin (KMO) value as 0.80 for the ESE-scale, the current study was also able to reproduce the acceptable KMO measure of sample adequacy (KMO = 0.819).

Thus, theoretically, ESE is significantly and positively correlated with students' laboratory performance. This positive correlation to students' laboratory performance was seen in Group 1 and Group 2, revealing ESE-scale's high validity. This result was supported by previous studies that self-efficacy has a strong significant effect on academic achievement (Fallan and Opstad, 2003). The linear regression analysis results indicated that gender mediated the relationship of experimental self-efficacy with laboratory score in both Groups. This suggests that the relationship between experimental self-efficacy and laboratory performance may differ for individuals of different genders. The negative coefficient (*B* = –0.266) in Group 2 indicates that the effect of ESE on the laboratory score is weaker for males than for females, whereas the positive coefficient (*B* = 0.213) in Group 1 indicates a stronger effect for males than for females. No gender difference was observed in the direct relationship between the laboratory performance and participants in both Group 1 and Group 2. Our results support previous studies that found differences between genders in self-efficacy and academic performance (Vantieghem et al., 2014; Alghamdi et al., 2020; Kong et al., 2021) and contradict studies that have found that gender has no effect on self-efficacy and strategy use (Kıran and Sungur, 2012). The results also suggest that the year of study has different effects on ESE and laboratory scores, depending on the group of participants. Specifically, in one group, the year of study was found to have a significant influence on participants' ESE, indicating that as participants progressed through their studies, they became more confident in their ability to perform experiments. In the second group, the year of study had a significant influence on laboratory scores, suggesting that as participants progressed through their studies, the level of difficulty increased, which affected their laboratory performance. However, the effect of the year of study was not consistent across both groups, indicating that other factors may also be influencing these outcomes. It is possible that the differences between the two groups of participants, such as their level of experience or the specific practical experiments they were assigned, may have contributed to these divergent results. The Pearson correlation results (Table 5) suggest that there was a difference in the correlation between ESE and lab scores of Group 1 (0.312–0.445) and Group 2 (0.788–0.850), which could be attributed to the type of practical experiments conducted during the study. It is possible that the differences in the physical experiments and the level of interactivity between the two groups influenced their self-efficacy beliefs, which in turn affected their performance on the laboratory tasks. Group 1 may have had less interactive experiments, leading to lower self-efficacy beliefs and performance, whereas Group 2 may have had more interactive experiments, leading to higher self-efficacy beliefs and performance. Additionally, it is worth noting that in Group 2, 62% of the participants are females only 38% are males. The linear regression analysis suggest that in Group 2 females have stronger effect on ESE-laboratory score relationship than males. This gender difference may also contribute to the higher correlation results in Group 2 compared to Group 1, as research has shown that females tend to have higher self-efficacy beliefs in academic and scientific domains than males (Huang, 2013; Sachitra and Bandara, 2017). Therefore, the higher percentage of female participants in Group 2 might be another reason for the stronger relationship between ESE and laboratory scores observed in that group. However, further investigation and analysis is needed to determine the precise nature of this relationship, including factors such as the duration, difficulty level, and perceived relevance of the experimental tasks.

It's important to note that self-efficacy can change over time and can be influenced by various individual and environmental factors, such as performance experiences, feedback, and social support. So, creating a positive learning environment and promoting a growth mindset can help students of all genders to increase their self-efficacy in the academic field.

The experimental self-efficacy scale provides greater flexibility in that it can be used to assess self-efficacy for a wide range of laboratory tasks and concepts rather than being specific to chemistry education. It is more applicable to students in a virtual or online laboratory (Achuthan et al., 2020) setting or to students in a different type of laboratory, such as an engineering or computer science laboratory.

## 5. Conclusions

Researchers and instructors in science education can now more carefully evaluate self-efficacy in laboratory settings. The experimental self-efficacy scale gives them a short but valid tool for analyzing the self-efficacy of students in laboratories. In order for individuals to confidently undertake experimentation and have higher experimental self-efficacy in any science laboratory, they have to supplement themselves with adequate knowledge and understanding of both the theory and experimental procedures. A measurement tool assists in examining and quantifying learners' self-efficacy beliefs in laboratories and taking consequent corrective action. The present research has successfully carried out a validation study of such a necessary tool or experimental self-efficacy scale. This work has culminated in the validation of a reliable scale with four factors and 12 items. The reliability coefficient of the ESE scale was highly indicative of the fact that the target structure was measured accurately by the items in the scale. Additionally, the scale items also predicted the target structure in terms of construct validity. This work also verified the applicability of this scale to other disciplines beyond chemistry, such as physics and biology, that are laboratory intensive. Further, this research was done with multiple cohorts of students pursuing education in these three scientific disciplines. It would be beneficial to extend this study with more disciplines and validate the findings.

## Data availability statement

The raw data supporting the conclusions of this article will be made available by the authors, without undue reservation.

## Ethics statement

The studies involving human participants were reviewed and approved by University Ethics Committee, Amrita Vishwa Vidyapeetham. The participants provided their written informed consent to participate in this study.

## Author contributions

VK and KA conceived and designed the study and wrote the manuscript. VK and SP collected the data. VK, SP, and KA revised the manuscript. All authors contributed to the article and approved the submitted version.

## Supplementary material

Supplementary Data Sheet Experimental self-efficacy scale.
