# Supplementary material for: Confirmatory and validation studies on experimental self-efficacy scale with applications to multiple scientific disciplines
Source: Front Psychol. 2023 Apr 18;14:1154310. doi: 10.3389/fpsyg.2023.1154310 (PMC10151821; doi:10.3389/fpsyg.2023.1154310)
Supplement: Supplementary Data Sheet — Experimental self-efficacy scale. [file Data_Sheet_1.PDF]

Experimental Self-efficacy (ESE) Questionnaire

No	Item	Factor
1	I believe I have a sound grasp of the theory behind laboratory experiments before performing experiments.	Conceptual Understanding (CU)
2	Experimental concepts become clearer to me as I perform the experiment.	
3	I am confident that I understand the underlying chemical phenomena in the experiment.	
4	I can usually handle the glass apparatus in the laboratory on my own without any fear of breakage and injury.	Laboratory Hazards (LH)
5	I am confident of working in the laboratory without chemical spillage.	
6	I am always alert in the laboratory and have minimal accidents.	
7	After an experiment, I have no difficulty figuring out how my calculation procedures and errors affected my results.	Procedural Complexity (PC)
8	When presented with laboratory results, I know how to interpret them and draw relevant conclusions from them.	
9	I do not struggle with processing information in background articles and relating them to my own laboratory procedures and results.	
10	I find it easy to complete the exercise in the laboratory even though there is limited personal participation in performing experiments.	Sufficiency of Resources (SR)
11	It is easy for me to understand theory and concepts properly in spite of limited availability of physical instruments.	
12	I do not find it challenging to understand an experiment even if there is only one try due to limited availability of chemicals.	

Reference: Kolil, Vysakh Kani, Sharanya Muthupalani, and Krishnashree Achuthan. "Virtual experimental platforms in chemistry laboratory education and its impact on experimental self-efficacy." *International Journal of Educational Technology in Higher Education* 17, no. 1 (2020): 1-22.
